# Multi-Bit Biomemristic Behavior for Neutral Polysaccharide Dextran Blended with Chitosan

**DOI:** 10.3390/nano12071072

**Published:** 2022-03-24

**Authors:** Lei Li

**Affiliations:** 1 HLJ Province Key Laboratories of Senior-Education for Electronic Engineering, Heilongjiang University, Harbin 150080, China; lileidtk@hlju.edu.cn; Tel.: +86-451-8660-8504; 2 Research Center for Fiber Optic Sensing Technology National Local Joint Engineering, Heilongjiang University, Harbin 150080, China

**Keywords:** neutral polysaccharide, biomemristance, dextran, proton conduction

## Abstract

Natural biomaterials applicable for biomemristors have drawn prominent attention and are of benefit to sustainability, biodegradability, biocompatibility, and metabolism. In this work, multi-bit biomemristors based on the neutral polysaccharide dextran were built using the spin-casting method, which was also employed to explore the effect of dextran on the ternary biomemristic behaviors of dextran–chitosan nanocomposites. The doping of 50 wt% dextran onto the bio-nanocomposite optimized the ratio of biomemristance in high-, intermediate-, and low-resistance states (10^5^:10^4^:1). The interaction between dextran and chitosan (hydrogen-bond network) was verified by Fourier transform infrared (FTIR) and Raman spectroscopy analysis; through this interaction, protons derived from the self-dissociation of water may migrate under the electric field, and so proton conduction may be the reason for the ternary biomemristic behaviors. Observations from X-ray diffraction (XRD), thermogravimetric analysis (TGA), and differential scanning calorimetry (DSC) analysis displayed that the 50 wt% dextran/50 wt% chitosan nanocomposite had the greatest amorphous ratio as well as the highest decomposition and peak transition temperatures in comparison with the other three dextran–chitosan nanocomposites. This work lays the foundation for neutral biomaterials applied to green ultra-high-density data-storage systems.

## 1. Introduction

Biopolymers have been regarded as one of the most promising resources for the fabrication of hybrid biomaterials, on account of their biocompatibility, biodegradability, ease of solubility, and hydrophilicity, coupled with various chemical functionalities [1]. As an attractive, versatile, and inexpensive combinational method, polymer blending can create novel bio-nanocomposites with tailored properties for biomemory applications and without external crosslinkers that can hamper biological activity [2,3]. Biopolymers, such as starch [4], cellulose [5], chitosan [6], and dextran [7], are commonly utilized in bio-nanocomposites. Notably, biodegradable films consisting of biopolymer materials have been proposed for application in drug-delivery systems, biomedicine, hydrogels, water treatment, food packaging, and membranes [8,9]. Natural polysaccharides such as chitosan and dextran are widely employed for biomedical applications [10]. Different methods have been used to fabricate dextran/chitosan-based composites as drug-loading and -delivery systems with therapeutic potential [11,12]. The good biocompatibility and functionality of both of these polymers, together with their biodegradability and nontoxic degradation byproducts, displays a great potential for incorporation with various active agents, including drugs and biologics.

Memristors play a promising role in building neuromorphic computing platforms, as they are accessible to the bottleneck of traditional von Neumann architecture [13]. Organic memristors bear the unique advantage of flexibility, low-temperature processability, and diverse functionalities, having enormous potential in biodegradable artificial neuromorphic systems [14,15]. Mostly, they are made up of undecomposable, biologically incompatible, and sometimes even toxic materials, causing serious ecological challenges [16,17,18]. Hence, it is significant that biodegradable and renewable materials should be exploited so as to realize green ultra-high-density data-storage systems. With the incremental consumption of nonrenewable resources and the growth rate of electronic waste, biodegradable electronic devices have seized considerable opportunities in advanced sustainable electronics [19]. Neutral natural biomaterials such as dextran, pullulan, and konjac flour are abundant in nature, and their biodegradability, nontoxicity, and renewability make for developing eco-friendly biodegradable electronics [20]. Nevertheless, biomemristors based on neutral natural biomaterials are rarely reported, and the research on their proton conductive behavior is as yet lacking. Thus, revealing the proton conduction mechanism of such materials can guide the working principles of biomemristors based on neutral natural biomaterials.

In this regard, an investigation of polysaccharide–based bio-nanocomposites was conducted with a focus on the biomemristic behaviors of dextran-based biomemristors. The solution-processable neutral-polysaccharide-dextran-based nanocomposite film acted as an active layer, for which remarkable ternary biomemristic behaviors were observed. Dextran is a nontoxic and biodegradable natural polysaccharide that is employed in the medical domain as a blood substitute, a polymeric carrier in the delivery of drugs, a plasma expander, and for bone healing [21]. Its molecular structure with excellent hydrophilicity contributes to the formation of a hydrogen-bond network and supplies a large number of proton transfer sites [22]. The neutral polysaccharide dextran, with proton conductive properties, provides a meaningful direction for biomemristors based on neutral polymers and advances the development of neutral natural biomaterials in biodegradable neuromorphic systems. Chitosan, an abundant, nontoxic, biodegradable, and biocompatible polymer, was selected due to its electronic insulation and proton conduction properties, which mean that it serves as a proton reservoir [2,3]. In more detail, the investigation was carried out to prepare an indium–tin oxide (ITO)/50 wt% dextran, 50 wt% chitosan/Ni biomemristor, and its functionality was engineered to gain a comparable memory performance in terms of low resistive switching voltages, excellent endurance, and retention properties that make a big step towards dextran-based biopolymers for the development of memory, logic, and computing devices.

## 2. Materials and Methods

### 2.1. Biomemristor Fabrication

Dextran (molecular formula (C_6_H_10_O_5_)_n_) with a molecular weight (M_W_) of 40 kDa was purchased from Sinopharm Chemical Reagent Co. Ltd., Shanghai, China, and used without any cleaning or treatment. Various amounts (x wt%) of carboxylated chitosan (molecular formula (C_6_H_11_NO_4_)_n_) (Aladdin, Shanghai, China) were dissolved in 20 mL of distilled water. After the chitosan solution was completely dissolved, (100-x) wt% of dextran powder was then added. The designations for nanocomposite systems are indicated in Table 1. The dextran–chitosan solution mixture (10 mg/mL) was stirred until a homogeneous solution was achieved, which was prepared by adding dextran into the chitosan solution at a weight ratio of 50 wt%, 25 wt%, 16.7 wt%, and 9.1 wt%, respectively. For a free-standing biomemristor, a glass substrate with an ITO layer (South China Science and Technology Company Limited, Shenzhen, China; surface resistance ≤6 Ω cm^−1^; transmissivity ≥ 84%; surface roughness R_a_ < 0.5 nm) was cleaned in acetone, absolute methanol, and absolute alcohol for 30 min by ultrasonication, followed by drying on a hot plate (Shanghai Bluepard Experimental Instrument Co., Ltd., Shanghai, China) at 40 °C. Next, the dextran–chitosan film as a dielectric layer was achieved through the spin-coating method at 3000 rpm/60 s and left to dry on a hot plate at 80 °C for 8 h. The spin-coating and annealing conditions were the same for all of the nanocomposite films ranging from B1 to B4. Consequently, Ni electrodes were evaporated onto the dextran–chitosan film using a shadow mask under 5 × 10^−4^ torr. All of the biomemristors were fabricated without packaging.

### 2.2. Dextran–Chitosan Nanocomposite Characterization

Different characterization techniques were utilized to confirm the formation of dextran–chitosan nanocomposites. XRD analyses were performed by X’PERT X-ray diffractometer (Panalytical Analytical Instruments Company, Almelo, Netherlands). The 2θ angle was varied from 5° to 70° (resolution of 0.01°). The degree of crystallinity (χ_c_) for dextran–chitosan nanocomposites was acquired as follows:

χ_c_ = A_c_/A_T_ × 100%, (1)

where the area of crystalline peak and total hump are denoted as A_c_ and A_T_, respectively. The areas of the peaks were determined via the Peak Analyzer technique in OriginPro9.1 software. XRD analyses were fitted in the 2nd derivative mode, where baseline correction and curve fitting were implemented to deconvolute them.

Fourier transform infrared (FTIR) spectroscopy was performed by a Foss DS 2500 Infrared Spectrometer (Foss NIRSystems Inc., Hillerød, Denmark) in the transmission mode (resolution of 1 cm^−1^), swept from 400 cm^−1^ to 4000 cm^−1^.

Raman spectroscopy (Horiba Jobin Yvon, Villeneuve-d’Ascq, France) was adopted to obtain the structure information of dextran–chitosan nanocomposites, scanned from 100 cm^−1^ to 3200 cm^−1^, with a 785 nm laser source and the power of the laser at 50 mW.

Thermogravimetric analysis (TGA) was conducted under N_2_ atmosphere by TA Instruments (New Castle, DE, USA) from 40 °C to 600 °C (heating rate of 10 °C/min). Differential scanning calorimetry (DSC) was performed by a NETZSCH DSC 3500 (Netzsch Scientific Instruments Trading Ltd., Selb, Germany). The samples were sealed in aluminum pans and heated from 40 °C to 250 °C (heating/cooling rate of 10 °C/min).

Electrical measurements of ITO/dextran–chitosan/Ni were fulfilled by a Keithley 4200 semiconductor parameter analyzer (Tektronix Inc., Solon, OH, USA) at room temperature. All of the electrical experiments were implemented without any device encapsulation in the air.

## 3. Results and Discussion

### 3.1. Determination of Dextran–Chitosan Nanocomposite Composition

**Figure 1:**
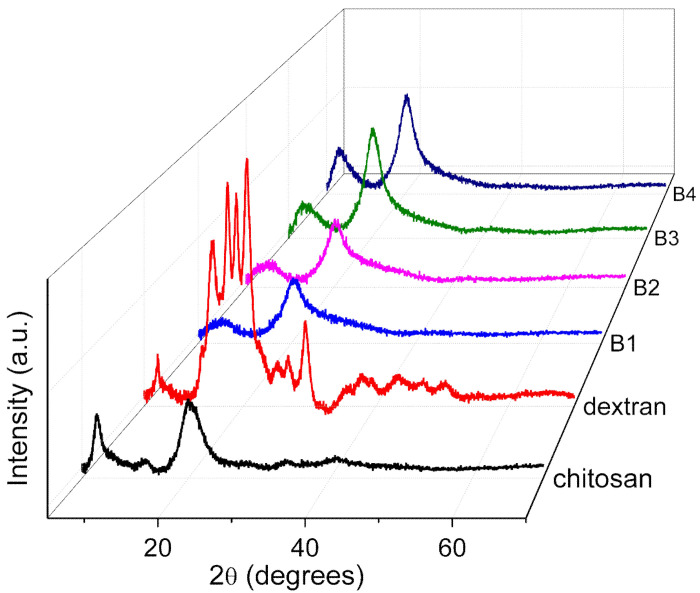
XRD patterns for chitosan, dextran, and nanocomposites (B1, B2, B3, and B4).

The XRD patterns (Figure 1) of the chitosan, dextran, and nanocomposites (B1, B2, B3, and B4) obviously revealed that the nanocomposite B1 took on fewer crystal peaks and a broad XRD pattern, so that B1 was the most amorphous nanocomposite. To confirm this observation, a Peak Analyzer was employed so that it was possible to extract the overlapping peaks of the amorphous and crystalline regions. The broad peaks are for the amorphous regions, while the sharp and small peaks are for the crystalline regions.

In the pattern of chitosan, two crystalline peaks were observed at 2θ = 7.3° and 13.9°, while one amorphous peak appeared at 2θ = 20.5° and 40.9°. Dextran bore crystalline peaks at 2θ = 7.0°, 15.5°, 17.7°, 19.0°, 20.5°, 22.2°, 25.7°, 26.8°, 29.4°, 35.3°, 38.3°, 43.6°, and 47.1°, and two amorphous peaks appeared at 2θ = 18.5° and 36.1°. The XRD patterns of the nanocomposites B1 and B4 were adopted to observe any changes in the peak position or intensity for the diffractograms of the nanocomposites. The intensity of the crystalline peaks in B1 was less than that in dextran. A crystalline peak at 13.9° in chitosan seemed to disappear, as portrayed in the XRD pattern of B1, when dextran was doped onto the nanocomposite. The XRD pattern of B1 presented that the intensity of two peaks at 2θ = 8.7° and 20.3° were restrained, which signified the interaction between dextran and chitosan [21]. In the XRD pattern of B4, the intensity of three peaks at 2θ = 8.0°, 20.3°, and 22.4° was incremental when more chitosan was added to the nanocomposite, illustrating that the amorphousness of B4 was diminished. The degree of crystallinity for B1, B2, B3, and B4 was calculated by Equation (1) and tabulated in Table 2. In detail, the nanocomposite B1 bore the lowest degree of crystallinity, which indicated that it was the most amorphous nanocomposite. Hence, the nanocomposite B1 was chosen as the polymer host for further analysis.

### 3.2. FTIR Analysis

**Figure 2:**
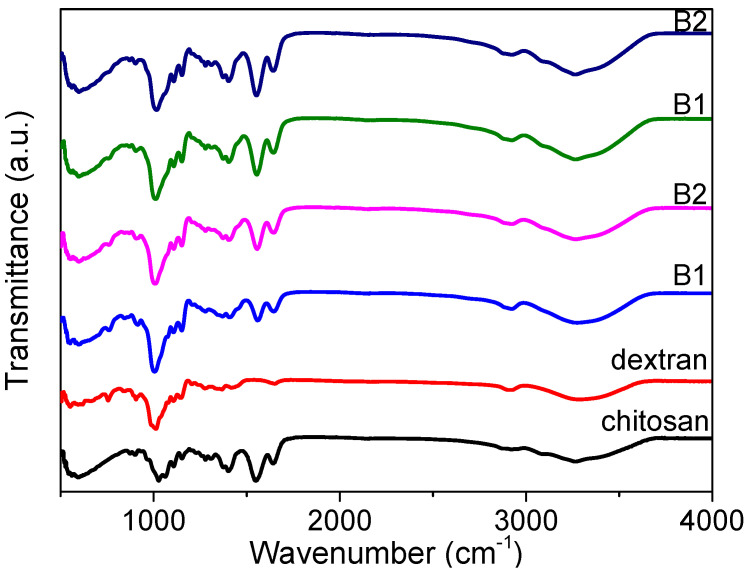
FTIR spectra for chitosan, dextran, and their nanocomposites.

FTIR spectroscopy measurements (Figure 2) for chitosan, dextran, and the nanocomposites (B1, B2, B3, and B4) were carried out in order to ascertain the interactions among the functional groups of the nanocomposites. The absorption spectrum displayed a strong and broad band at 3000–3700 cm^−1^, derived from the stretching vibration of hydroxyl groups (O-H) [23]. There was apparently a large variety of O-H groups in the dextran chains responsible for its good solubility in water. This does not favor the preparation of large-area films in a solution-processable method, but it does enhance the dextran-based biomemristor. It offers a great possibility for proton conduction as a result of the existence of the hydroxyl groups.

The FTIR analysis also portrayed the spectra of chitosan, dextran, and the nanocomposites (B1, B2, B3, and B4) in the glycosidic linkage (C-O-C), amine, and hydroxyl band regions [24]. The glycosidic linkage band peak for dextran arose at 905 cm^−1^, while the peak for chitosan was at 1027 cm^−1^. The glycosidic band peak was transformed from 903 cm^−1^ to 915 cm^−1^ when dextran was increasingly incorporated into the chitosan, while the amine band peak was shifted from 1552 cm^−1^ to 1558 cm^−1^. The amine band for chitosan was centered at 1550 cm^−1^, while there was no amine functional group for dextran. Moreover, the amine band peak was moved to 1558 cm^−1^ in the spectrum of B1, and the peak continued to shift to a lower wavenumber of 1552 cm^−1^ for B4. This is evidence of the interaction between the nitrogen atoms of the polymer matrix with the cations [21]. The broad hydroxyl band peak of dextran was located at 3288 cm^−1^, while that of chitosan was centered at 3264 cm^−1^. The position of the hydroxyl peak for B4 was similar to that for chitosan. This could result from a higher content of chitosan in the nanocomposite. The changes (Figure 2) in peak’s position, intensity, and shape illustrated that hydrogen bonding interactions occured in these regions [25,26,27]. This convincingly demonstrates the promising potential of the biomemristors for future multi-bit data-storage systems.

### 3.3. TGA Analysis

**Figure 3:**
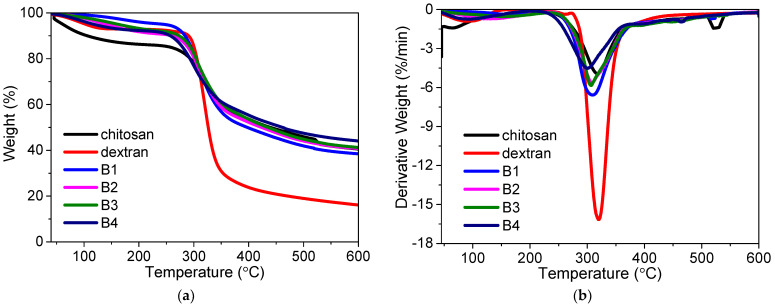
TGA–DTG of thermograms of chitosan, dextran, and their nanocomposites: (**a**) TGA and (**b**) DTG of thermograms for chitosan, dextran, and their nanocomposites.

The influence of polymer blending on the thermal stability could be ascertained by TGA–DTG measurements (Figure 3). It could be seen that chitosan, dextran, and their nanocomposites experienced three thermal degradation processes during the 40–600 °C heating treatment. The first decomposition in all the TGA thermograms from 40 °C to 170 °C was attributed to the water weight loss due to the polymer possesses’ hydroscopic nature [28]. The second decomposition for chitosan started at 287 °C and ended at 371 °C, while the dextran started to decompose at 300 °C and stopped decomposing at 348 °C. The essential mass loss stemmed from the decomposition of the polymeric chains, resulting in the rupture of C-C and C-O bonds. The third weight loss was ascribed to the formation of carbonaceous or polynuclear aromatic structures.

Dextran underwent single-step decomposition in the temperature range of 300–348 °C, with a DTG peak temperature of 320 °C. By blending the dextran with chitosan, the decomposition temperature (T_d_) appeared at 309 °C, 308 °C, 307 °C, and 301 °C for B1, B2, B3, and B4, respectively. The degradation temperatures of the thermal changes are given in Table 3. Fifty percent weight loss for chitosan and dextran was recorded at 439 °C and 326 °C, respectively. During the early stage of native polysaccharide degradation, there is a sudden drop in the thermogravimetric curve. This may be assigned to the elimination of bound water from dextran [25]. Dextran revealed a single-stage degradation, as the water content was low due to its hydrophobic nature. Fifty percent weight loss was reached at 398 °C, 424 °C, 435 °C, and 462 °C for the nanocomposites B1, B2, B3, and B4, respectively. Although the existence of distinct substituents in the para position of the aromatic ring makes it difficult to compare polymers’ thermal stability [29], an increase in the 50% weight loss temperature values was clearly observed with the increase in chitosan. An improvement in thermal stability after blending could also be seen for other polysaccharides, such as pullulan [30], starch [31], xylan [32], hemicelluloses, and glucomannan [33,34].

### 3.4. DSC Analysis

**Figure 4:**
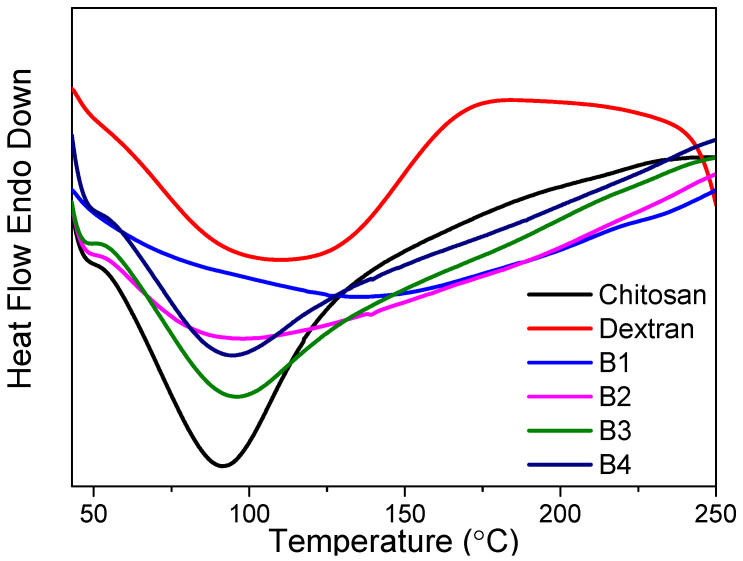
DSC thermograms of dextran, chitosan, and their nanocomposites.

DSC analysis (Figure 4) was implemented to ascertain the peak transition temperatures (T_p_) of the samples. The T_p_ is bound up with the breaking of the hydrogen bonds associated with the spatial conformation of dextran–chitosan nanocomposites. The higher the tT_p_ is, the higher the thermal stability and the more compact the formation of dextran–chitosan nanocomposites will be. The DSC analysis of dextran indicated an apparent endothermic peak at 110 °C, conforming to the T_p_ of dextran, but the T_p_ of the nanocomposite B4 obviously fell to 95 °C after chitosan incorporation (Table 3). The loose structure of the dextran–chitosan nanocomposites might account for this [25].

### 3.5. Raman Spectra

**Figure 5:**
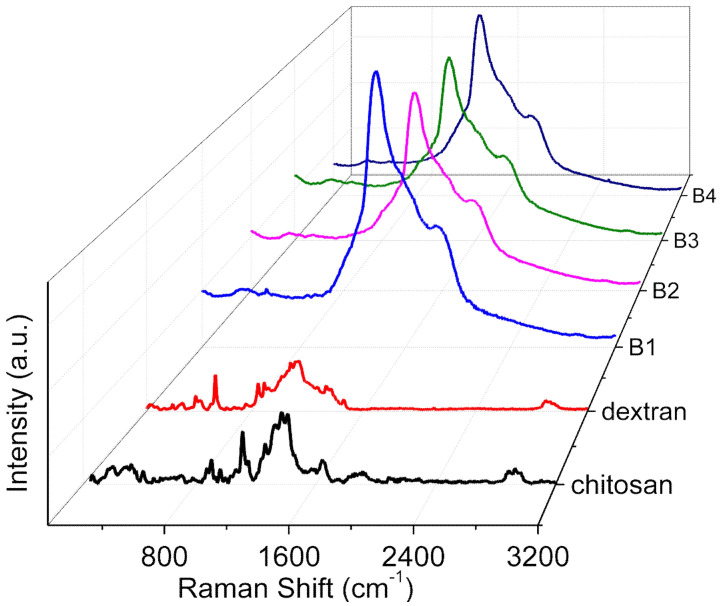
Raman spectra of chitosan, dextran, and their nanocomposites (B1, B2, B3, and B4).

**Figure 6:**
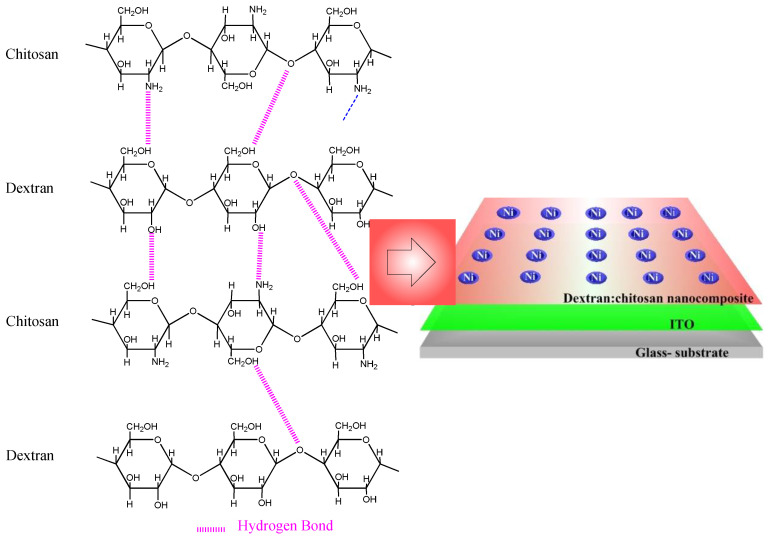
Schematic diagram of molecular structure of chitosan and dextran; interaction between chitosan and dextran; configuration of ITO/dextran–chitosan/Ni biomemristor.

Raman spectra (Figure 5) for the internal mode region of chitosan, dextran, and their nanocomposites (B1, B2, B3, and B4) were plotted in the solid state. The nature of the interaction between the two polymers and the appreciable structural changes (Figure 6) were elucidated. The peak at 542 cm^−1^ was in virtue of the vibrations of atoms with the participation of the C4-C5-O of the glucopyranose ring and the C1-O6-C6 of the glycoside bond. The bands at 850 cm^−1^ and 923 cm^−1^ occurred due to C-O-C stretching. With the incremental content of chitosan (B1, B2, B3, and B4), the band at 542 cm^−1^ had a low intensity, manifesting dextran and chitosan compatibility as a result of the interaction between the two [20].

For chitosan, a prominent peak at 1645 cm^−1^ originated from NH wagging and carbonyl stretching of the amide group. The augmentation of the dextran content up to 50% rendered the signal intensity extremely weak for the peak due to the glucopyranose ring and glycoside bond interaction, as well as for NH wagging, exhibiting the existence of a strong interaction between the two components [20]. By the comparison between pure chitosan and its nanocomposites (B1, B2, B3, and B4), a red shift in the peaks from 1380 cm^−1^ to 1369 cm^−1^ (ν(C-H) in (CH_2_OH)) as a result of hydrogen bonding interactions between chitosan and dextran could be seen [21]. The hydrogen-bond network between dextran and chitosan facilitated the highly proton-conductive property of the dextran–chitosan matrix.

### 3.6. Biomemristic Characteristics of ITO/Dextran–Chitosan/Ni

**Figure 7:**
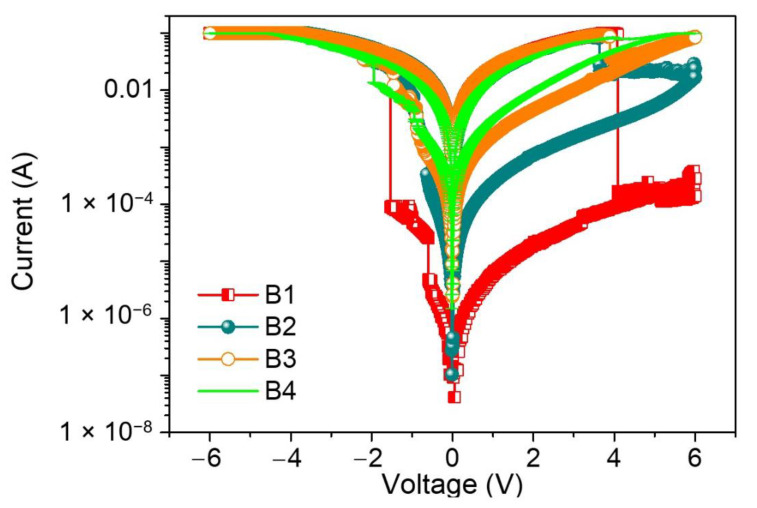
Current–voltage (I–V) characteristics of ITO/B1/Ni, ITO/B2/Ni, ITO/B3/Ni, and ITO/B4/Ni.

Dextran, a neutral polysaccharide, was first utilized in the dielectric layer to fabricate the ITO/dextran–chitosan/Ni biomemristor (Figure 6), while chitosan, an abundant, nontoxic, biodegradable, and biocompatible polymer, was selected owing to its electronic insulation and proton-conduction properties. Significantly, electronic systems taking account of this neutral polysaccharide could promote the recycling and management of waste streams, thereby being advantageous to our living environment. The memristor comprises a two-terminal device with a dielectric layer sandwiched between metal electrodes in a vertical configuration. The proposed hydrogen-bonding interaction between chitosan and dextran is illustrated according to the aforementioned characterization observations. The protons, hopping through the dielectric layer, behave like the ligands of biological cells. The mobile protons in the dielectric layer act as the memory layer and migrate in response to the bias. To demonstrate that the dextran-based biomemristors had the essential ability for biomemory functions, the electrical properties were tested at a bias sweeping between −6 V and +6 V (Figure 7). After the fabrication of the devices, the current–voltage (I–V) characteristics of the devices regarding B1, B2, B3, and B4 were investigated.

The I–V plots for ITO/B1/Ni displayed multi-bit biomemristic behaviors with tristable resistive switching (RS). Under an applied bias voltage from 0 V to −6 V (sweep 1), the current flowed initially in a high resistive state (HRS), but the current suddenly grew to another state—an intermediate resistive state (IRS) at a set voltage of V_SET1_ = −0.60 V. During the continuous scanning, the current switched again to a low resistive state (LRS) at a set voltage of V_SET2_ = −1.54 V. These two sudden current increases stood for the “writing” process in the biomemristor. Then, LRS was kept until the next scan (sweep 2). However, the current abruptly fell up to a reset voltage of V_RESET_ = 4.09 V when scanning in reverse from 0 to +6 V (sweep 3). This sudden current transition from LRS to HRS was treated as the “erasing” process. A dozen of the two-terminal devices were tested and over 90% showed such a ternary data storage capability.

To further investigate the effect of dextran on biomemristors, the current was tested on different nanocomposites. Interestingly, as the blending concentration of dextran decreased from 50 wt% to 9.1 wt%, the devices presented similar tristable RS behaviors to the ITO/B1/Ni device. As the content of dextran decreased, a lower R_HRS_:R_IRS_:R_LRS_ was observed under the voltage sweeping. The tristability of the RS properties was drastically tuned by dropping the dextran content to 25 wt%. There were two sudden current increases at V_SET1_ = −0.64 V and V_SET2_ = −0.96 V, transiting from HRS to IRS and from IRS to LRS. Moreover, LRS was abruptly switched to HRS at V_RESET_ = 3.64 V without the appearance of IRS. For ITO/B3/Ni and ITO/B4/Ni, V_SET1_, V_SET2_, and V_RESET_ were −0.8 V, −1.5 V, and 4 V and −1.1 V, −2.45 V, and 4 V, respectively. In addition, there was no RS observed in the I–V curves of the devices with dextran or chitosan as the active material. That is to say, the dextran blending may provide much more efficient trapping sites than the homogenously dispersed chitosan. These observations guarantee that the blending content of dextran plays an essential role in the ternary biomemristic behaviors of the dextran–chitosan devices.

**Figure 8:**
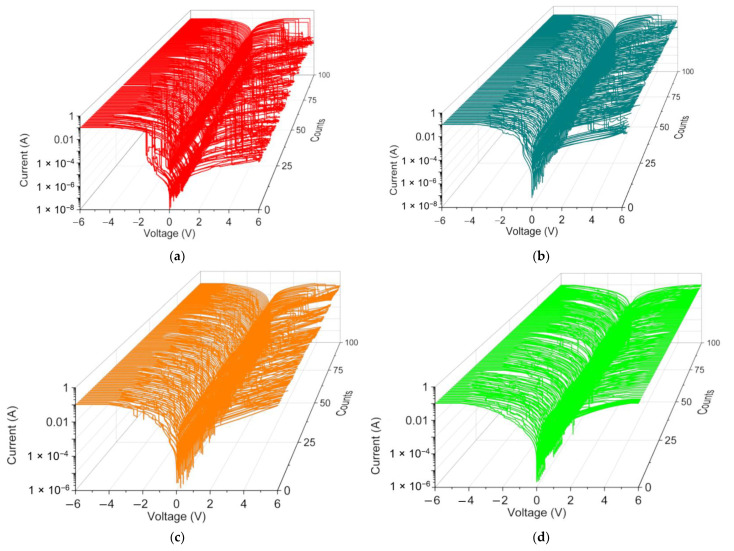
Ternary I–V characteristics of (**a**) ITO/B1/Ni, (**b**) ITO/B2/Ni, (**c**) ITO/B3/Ni, and (**d**) ITO/B4/Ni for 100 consecutive cycles in DC double logarithmic modes.

**Figure 9:**
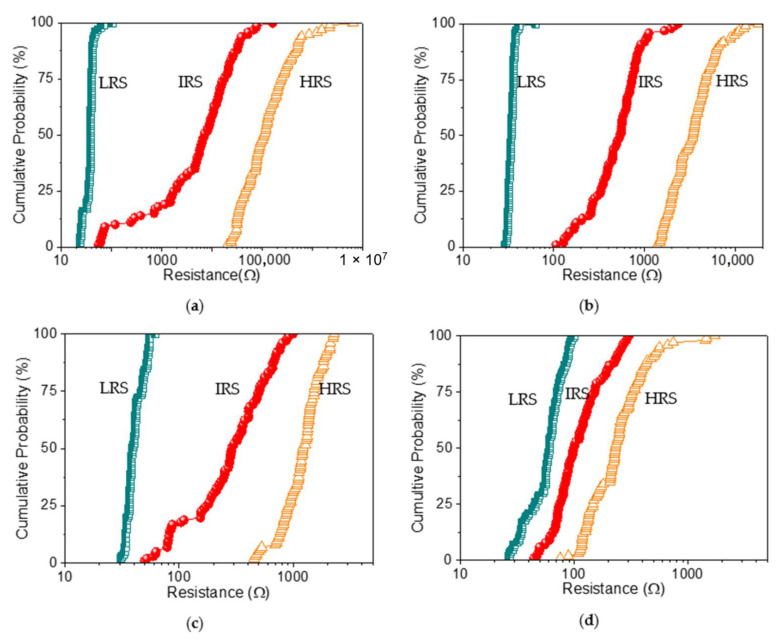
Cumulative probability of the resistance values in LRS, IRS, and HRS of (**a**) ITO/B1/Ni, (**b**) ITO/B2/Ni, (**c**) ITO/B3/Ni, and (**d**) ITO/B4/Ni for 100 consecutive cycles in DC double logarithmic modes.

The switching cyclability (endurance) analysis of the dextran-based biomemristor (Figure 8) clearly presented the multi-bit data storage feature with a distinguishable number of levels and then plotted this as cumulative analyses in order to better understand the resistance distribution (Figure 9). The distribution of the resistance in HRS, IRS, and LRS (R_HRS_, R_IRS_, and R_LRS_) suggested that R_LRS_ was tightly distributed in the measured resistance, while R_HRS_ and R_IRS_ held a loose distribution. These RS cycles were maintained even after cycle-to-cycle scanning (up to 100 times). Most strikingly, the variation of the mean resistance in HRS, IRS, and LRS according to the distinct weight ratio of chitosan and dextran (Table 4) revealed that the average resistance ratio R_HRS_:R_IRS_:R_LRS_ for ITO/B1/Ni was roughly 10^5^:10^4^:1. It is important to note that the device with the B2 system exhibited a lower R_HRS_:R_IRS_:R_LRS_ ratio (10^3^:10^2^:1). The 9.1 wt% dextran-based device displayed the lowest resistance ratio (5:2:1) compared with that of the 16.7 wt% dextran-based biomemristor (30:10:1). With the incremental increase in the content of dextran from 9.1 wt% to 50 wt%, a significantly enhanced trend was observed in the R_HRS_:R_IRS_:R_LRS_ ratio for the dextran–chitosan devices, as the multi-bit memory behavior was available. The indication was that the introduction of dextran played a critical role in enhancing the R_HRS_:R_IRS_:R_LRS_ of the devices, particularly by reducing the current level in R_HRS_ and R_IRS_ while increasing the current level in R_LRS_. 

**Figure 10:**
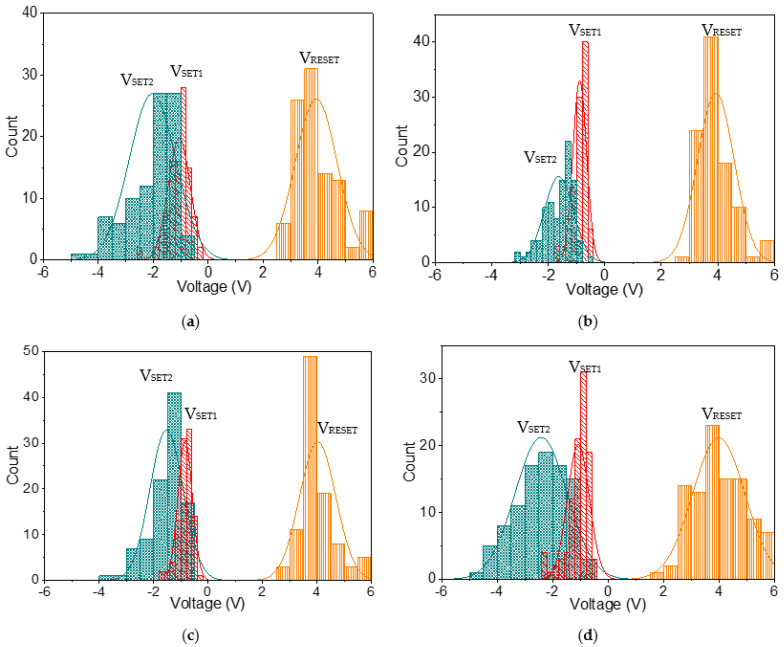
Histogram curves of V_SET1_, V_SET2_, and V_RESET_ for (**a**) ITO/B1/Ni, (**b**) ITO/B2/Ni, (**c**) ITO/B3/Ni, and (**d**) ITO/B4/Ni during repeated resistive switching.

Histogram analyses with normal fitting lines for V_SET1_, V_SET2_, and V_RESET_ for ITO/B1/Ni (Figure 10a) were plotted, ranging from −0.26 V to −2.6 V, from −0.77 V to −4.74 V, and from 2.51 V to 5.96 V, respectively; the corresponding central values were −1.05 V, −2 V, and 3.85 V, respectively. In the statistical data of ITO/B2/Ni (Figure 10b), V_SET1_, V_SET2_, and V_RESET_ ranged from −0.52 V to −1.77 V, from −0.59 V to −3.19 V, and from 2.99 V to 6 V, respectively, and their central voltages were located at −0.9 V, −1.6 V, and 3.93 V, respectively. In the histogram analyses of ITO/B3/Ni (Figure 10c), the SET1, SET2, and RESET voltages were observed in the range −0.4 V to −1.79 V, −0.67 V to −3.55 V, and 2.94 V to 6 V, respectively, and the central values were −0.8 V, −1.5 V, and 4 V, respectively. Moreover, the SET1 and SET2 voltage of ITO/B4/Ni (Figure 10d) arose between −0.65 V and −2.4 V and between −0.67 V and −4.65 V, while the RESET voltage occurred between 1.97 V and 5.66 V. Their magnitudes mostly focused on −1.1 V, −2.45 V, and 4 V. Additionally, the statistical data revealed that the SET1, SET2, and RESET voltages were quite stable, showing relatively little deviation from the average value (Table 5). This indicates that these results are highly reproducible for the tristable RS.

**Figure 11:**
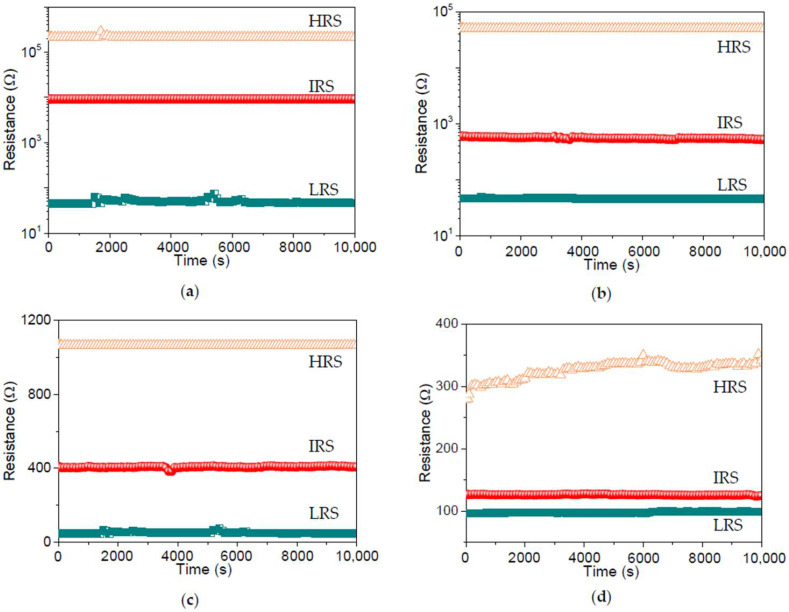
Retention tests conducted at R_HRS_, R_IRS_, and R_LRS_ of (**a**) ITO/B1/Ni, (**b**) ITO/B2/Ni, (**c**) ITO/B3/Ni, and (**d**) ITO/B4/Ni without evident changes for over 10^4^ s.

To assess the stability of the dextran-based biomemristic devices, the retention ability was measured by applying a constant voltage (0.1 V) to the top electrode. Furthermore, the retention tests (Figure 11) for ITO/B1/Ni, ITO/B2/Ni, ITO/B3/Ni, and ITO/B4/Ni suggested that R_HRS_, R_IRS_, and R_LRS_ had no noticeable degradation during the retention tests, with excellent nonvolatile retention properties over 10^4^ s.

Overall, the multi-bit and nonvolatile characteristics of the dextran-based biomemristor make it suitable for conducting ultra-high-density data-storage applications.

### 3.7. Operational Mechanism of Biomemristor

It is also interesting to explore the tristable RS mechanisms of the dextran–chitosan devices, which were analyzed by the linear fitting of the measured I–V plots. To throw light on the current conduction mechanism, the space-charge-limited conduction (SCLC) theory was invoked, and the I–V characteristics were analyzed by the power law [35]:

I ∝ V^α^, (2)

where α is the scaling exponent bound up to the depth of the trap state distribution under the conduction band. Dependent on the power law, α = 1 conforms to the Ohmic regime (I ∝ V), where the applied bias is not strong enough to eject electrons from the electrodes and the conduction is governed by only the thermally generated electrons. In contrast, α = 2 brings about a square-law dependence (I ∝ V^2^), illustrating that trap states are partially filled by electrons and the current conduction is governed by the hopping of electrons through the trap states. On the other hand, α > 2 corresponds with trap-filled limited conduction, where the trap states become completely filled and a smooth electronic transport happens. Thus, it turns into a trap-free state and the possible current conduction mechanism is trap-controlled space-charge-limited conduction. In this case, trap states are realized by the presence of dextran–chitosan nanocomposites. Additionally, the migration of protons plays a significant role in the current conduction.

**Figure 12:**
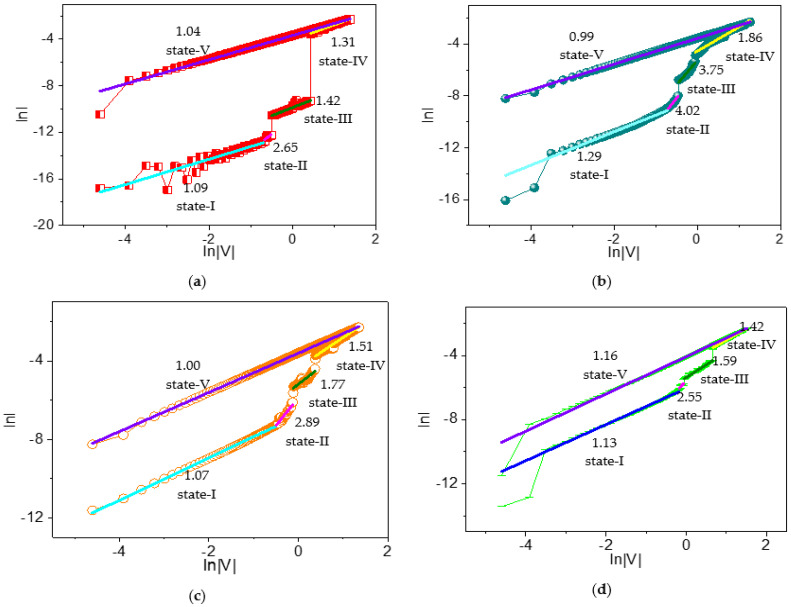
Log–log plots of the I-V data for (**a**) ITO/B1/Ni, (**b**) ITO/B2/Ni, (**c**) ITO/B3/Ni, and (**d**) ITO/B4/Ni during the SET process.

To understand the current conduction mechanism in dextran-based biomemristors, reverse biased I–V curves were plotted on a logarithmic scale (Figure 12). Moreover, the slopes of different regions were calculated and depicted. The scaling exponent parameter α could be extracted from the slope of the linearly fitted log–log I–V plots. In the linear fitting curves of the log–log I–V for ITO/B1/Ni, state I with a slope of −1.09 suggested an Ohmic-like conduction, while state II with a slope of 2.65 revealed a trap-free current conduction at higher voltages under HRS. In contrast, state III (slope = 1.42) showed Ohmic-like conduction under IRS. After that, state IV (slope = 1.31) showed Ohmic-like current conduction at higher voltages. During LRS, state V (slope = 1.04) showed Ohmic-like conduction.

Likewise, the possible current conduction mechanism of ITO/B2/Ni was addressed by the linear curve fitting of I–V plots on a logarithmic scale, and different regimes were identified by applying the power law. State I (slope = 1.29), state II (slope = 4.02), state III (slope = 3.75), and state IV (slope = 1.86) corresponded to Ohmic, trap-free, trap-free, and trap-filled limited current conduction, respectively. For LRS, fitting a linear curve of state V displayed its slope (0.99). The ITO/B3/Ni device similarly demonstrated an Ohmic-like conduction at lower voltages (slope = 1.07) and a trap-free current conduction mechanism at higher voltages (slope = 2.89) for HRS under reverse bias. For IRS under reverse bias, it was observed to be trap-fill limited conduction (slope = 1.77). For LRS, a condition slope = 1 gave rise to Ohmic-like current conduction. Analogously, state I (slope = 1.13), state II (slope = 2.55), state III (slope = 1.59), state IV (slope = 1.42), and state V (slope = 1.16) of ITO/B4/Ni conformed to Ohmic, trap-free, trap-free, trap-filled limited, Ohmic, and Ohmic current conduction, respectively.

Therefore, the I–V relationship of the dextran-based ternary biomemristors in HRS contained two different conductive regions, as follows: (i) a low-voltage region conforming to the Ohmic conduction mechanism, and (ii) a high-voltage region associated with trap-free current conduction. The biomemristic behaviors for the devices in IRS and LRS were in accordance with the trap-free current conduction and Ohmic conduction mechanisms of SCLC.

Dextran is a neutral polysaccharide without dissociating protons from its molecular chain. Its molecular structure, with excellent hydrophilicity, facilitates the formation of a hydrogen-bonding network and provides a large number of proton transfer sites [22]. As a matter of fact, water molecules inevitably remain in the dextran film as a result of the solution preparation and its hydroxyl groups. Hence, protons may come from the self-dissociation of water, and the highly proton-conductive property of the dextran–chitosan matrix stems from the hydrogen-bond network between dextran and chitosan. After a bias sweeping process, the protons were successively accumulated at the nanocomposite/electrode interface and the chitosan/dextran interface [22,36]. From the slopes of the I–V curves in state I and II, the current changes were based on low-exponent space-charge-limited current (SCLC) as the voltage increased. Thus, the resistance (HRS) was relatively large. The injection of more protons would fill the trapping sites, with a further increase in the voltage, leading to a dramatic growth in resistance (IRS). As the voltage rises, protons captured at the trapping sites diminish the existing trap concentration, and the resistance (LRS) decreases. As a consequence, an obvious tristable RS is generated by proton migrating in the dextran–chitosan nanocomposite film. Therefore, the physical mechanism for the biomemristic behaviors of ITO/dextran–chitosan/Ni is predominantly attributed to the SCLC theory and results from the charge trapping/detrapping process with different filling ratios of protons in the trapping sites. The increase in dextran contributes to a higher level of water absorption in the dextran–chitosan film, which can produce more proton-conducting hydrogen bond chains that serve as proton wires for Grotthuss-type transfer [22].

The ability of the dextran–chitosan film to immobilize and accommodate a large number of protons guarantees the good retention of the device and lays the foundation for an ultra-high-density memory capacity.

## 4. Conclusions

This feasible strategy to explore the biomemristic behaviors of a neutral natural polysaccharide in biomemristors was the first attempt to demonstrate such a device and revealed remarkably good endurance (>10^2^ times) and retention properties (10^4^ s) based on proton conduction. Multi-bit biomemristors were developed by means of the tristable RS of dextran and chitosan induced by proton migration. They possessed the required low switching voltages that resulted in a lower power dissipation during the RS process, when the biomemristors could degrade without any toxic or harmful byproducts. In light of a sustainable future, neutral natural materials could become a promising alternative to current expensive, nondegradable or non-biocompatible electronic devices, and they possess enormous application potential in “green” multi-bit data storage. The design and implementation of dextran–chitosan nanocomposite films will also highlight them as emerging environmentally friendly materials used for neuromorphic bioelectronics.

## Figures and Tables

**Table 1:** Designation for dextran–chitosan nanocomposite systems.

Dextran–Chitosan Composition (wt%)	Designation
50 wt%, 50 wt%	B1
25 wt%, 75 wt%	B2
16.7 wt%, 83.7 wt%	B3
9.1 wt%, 90.9 wt%	B4

**Table 2:** Degree of crystallinity for the nanocomposites B1, B2, B3, and B4.

Polymer Nanocomposite	Degree of Crystallinity (χ_c_)
B1	12.40%
B2	14.11%
B3	24.64%
B4	22.09%

**Table 3:** Decomposition and peak transition temperatures of chitosan, dextran, and their nanocomposites.

Sample	T_d_ (°C)	T_p_ (°C)
Chitosan	318	92
Dextran	320	110
B1	309	136
B2	308	98
B3	307	96
B4	301	95

**Table 4:** Average resistance value in the high resistive state, intermediate resistive state, and low resistive state (R_HRS_, R_IRS,_ and R_LRS_) for cycle-to-cycle analysis.

Sample	R_HRS_ (kΩ)	R_IRS_ (kΩ)	R_LRS_ (Ω)	R_HRS_:R_IRS_:R_LRS_
B1	288.13	14.16	37.60	10^5^:10^4^:1
B2	4.02	0.58	34.34	10^3^:10^2^:1
B3	1.28	0.36	41.62	30:10:1
B4	0.29	0.12	59.99	5:2:1

**Table 5:** Average of V_SET1_, V_SET2_, V_RESET_ for cycle-to-cycle analysis.

Sample	V_SET1_ (V)	V_SET2_ (V)	V_RESET_ (V)
B1	−1.06	−2.03	3.93
B2	−0.89	−1.63	3.93
B3	−0.85	−1.53	4.02
B4	−1.08	−2.43	3.97

## Data Availability

The data presented in this study are available on request from the corresponding author.
